# An international consensus on the essential and desirable criteria for an ‘organized’ cancer screening programme

**DOI:** 10.1186/s12916-022-02291-7

**Published:** 2022-03-23

**Authors:** Li Zhang, André L. Carvalho, Isabel Mosquera, Tianmeng Wen, Eric Lucas, Catherine Sauvaget, Richard Muwonge, Marc Arbyn, Elisabete Weiderpass, Partha Basu

**Affiliations:** 1 International Agency for Research on Cancer, Lyon, France; 2 National Cancer Center/National Clinical Research Center for Cancer/Cancer Hospital, Chinese Academy of Medical Sciences and Peking Union Medical College, Beijing, China; 3 Unit of Cancer Epidemiology, Belgian Cancer Centre, Sciensano, Brussels, Belgium; 4 Department of Human Structure and Repair, Faculty of Medicine and Health Sciences, University Ghent, Ghent, Belgium

**Keywords:** Organized cancer screening programme, Delphi, Consensus, Essential criteria, Desirable criteria

## Abstract

**Background:**

High variability in the definition and interpretation of organized cancer screening needs to be addressed systematically. Moreover, the relevance of the current practice of categorizing screening programmes dichotomously into organized or non-organized needs to be revisited in the context of considerable heterogeneity that exists in the delivery of cancer screening in the real world. We aimed to identify the essential and desirable criteria for organized cancer screening that serve as a charter of best practices in cancer screening.

**Methods:**

We first did a systematic review of literature to arrive at an exhaustive list of criteria used by various publications to describe or define organized cancer screening, based on which, a consolidated list of criteria was generated. Next, we used a Delphi process comprising of two rounds of online surveys to seek agreement of experts to categorize each criterion into essential, desirable, or neither. Consensus was considered to have been achieved based on a predetermined criterion of agreement from at least 80% of the experts. The outcomes were presented before the experts in a virtual meeting for feedbacks and clarifications.

**Results:**

A total of 32 consolidated criteria for an organized screening programme were identified and presented to 24 experts from 20 countries to select the essential criteria in the Delphi first round. Total 16 criteria were selected as essential with the topmost criteria (based on the agreement of 96% of experts) being the availability of a protocol/guideline describing at least the target population, screening intervals, screening tests, referral pathway, management of positive cases and a system being in place to identify the eligible populations. In the second round of Delphi, the experts selected eight desirable criteria out of the rest 16. The most agreed upon desirable criterion was existence of a specified organization or a team responsible for programme implementation and/or coordination.

**Conclusions:**

We established an international consensus on essential and desirable criteria, which screening programmes would aspire to fulfil to be better-organized. The harmonized criteria are a ready-to-use guide for programme managers and policymakers to prioritize interventions and resources rather than supporting the dichotomous and simplistic approach of categorizing programmes as organized or non-organized.

**Supplementary Information:**

The online version contains supplementary material available at 10.1186/s12916-022-02291-7.

## Background

It is well-accepted that the implementation of cancer screening with an ‘organized’ approach can significantly reduce mortality from the cancers targeted for population screening (e.g. cervical, breast and colorectal cancer) [1–4]. Organized screening is expected to enhance screening participation, decrease disparities, and minimize the harms of screening by ensuring high quality of services across the entire care continuum and reducing over-screening. According to the World Health Organization (WHO), only when implemented through an organized approach, screening programmes are likely to achieve a high coverage of the at-risk population and deliver desired impact at the population level [5]. Organized screening programmes spend healthcare resources in a more cost-effective manner [6].

Organized cancer screening, as traditionally interpreted, is a resource-intensive public health activity requiring substantial investments into health service infrastructure, human resources, information system and quality assurance [7]. Such investments require strong political commitment and thorough understanding of principles of organization of high-quality screening services. Despite the frequent attempts to label screening programmes as organized or not, there is considerable lack of clarity about the criteria that a screening programme needs to fulfil to be labelled as ‘organized’. In absence of an international consensus, the term has often been used interchangeably with ‘population-based screening’ defined as a programme that systematically invites the eligible population to be screened [8, 9]. While being population-based is a well-recognized criterion of organized screening, the latter requires many other conditions to fulfil. In fact, the response to the question of whether a programme is organized may not be a simple binary ‘yes’ or ‘no’. Programme organization requires compliance to a range of pre-conditions that need to be defined and a consensus be achieved. The need for an explicit delineation of the characteristics of an organized screening programme has been expressed before [10]. Enumeration of the criteria for screening programme organization and their categorization into ‘essential’ and ‘desirable’ are expected to provide guidance to cancer screening programmes managers and policymakers to prioritize services and allocate resources. Moreover, such an exercise will help compare status and performance of different programmes.

Our present study used a systematic review to identify the various criteria listed in the literature. Due to considerable heterogeneity in the definition and the practice of organized cancer screening, Delphi consultation is needed to receive the opinion of the experts in a structured manner and further to arrive at a consensus on the essential and the desirable criteria of organized screening.

## Methods

The study was conducted by the International Agency for Research on Cancer (IARC/WHO) as a blend of a systematic review and an online modified Delphi process. The study was carried out in three phases between 05/2020 and 09/2021. In the first phase, a systematic review of literature was performed to identify and synthesize the criteria for cancer screening programme organization used by different guidelines, expert opinions, and research reports. This was followed by two successive rounds of online surveys to seek international experts’ agreement on essential and desirable criteria for an organized cancer screening programme. Finally, a virtual meeting of the experts participating in the surveys was held to present the outcomes, receive their feedbacks and resolve any pending issues. The details of each step are described in the following sections.

### Systematic review

**Fig. 1:**
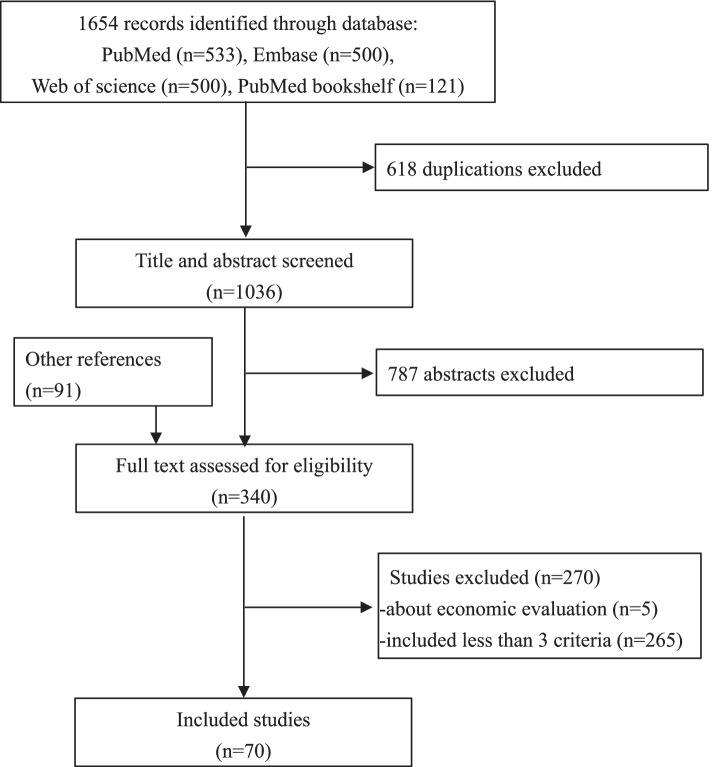
PRISMA flow diagram of the study selection process

PRISMA flow diagram of the study selection process is shown in Fig. 1. A systematic literature retrieval was conducted using the bibliographic search engines of PubMed, Embase, Web of Science and PubMed bookshelf to identify criteria used in previous publications that describe or define organized cancer screening. All databases were searched from inception to 04/09/2020 without any language limitation. Additional pertinent references and grey literature were requested from members of the cancer screening expert group (described later) to complement the list of selected published articles. The search strategies used in different databases, inclusion and exclusion criteria are described in the Additional file 1: Box S1. To be eligible the studies had to list at least three criteria for organized programmes.

Screening of the titles and abstracts followed by full-text review was independently conducted by two investigators (LZ, TMW). All cancer screening organization related criteria were directly copied from the literature to a table. The criteria that were exact duplicates were removed and the table containing 69 individual criteria was shared with two senior investigators (PB, AC) for independent review. Based on the raw extracted information, each senior investigator consolidated the grossly overlapping ones into a smaller number of criteria independently. If there was a disagreement between PB and AC about whether some criteria could be considered as overlapping, all were retained in the list.

### Creation of an expert group

An expert group representing diverse disciplines related to cancer screening and different resource settings was formed through a systematic mapping exercise. Experts involved in planning, implementation and evaluation of cancer screening programmes and related research from all around the world are members of an advisory board of the ‘Cancer Screening in Five Continents’ (CanScreen5) project of IARC [11]. An initial list of experts was prepared from those empanelled in CanScreen5 project with representation from various geographic areas, healthcare settings and disciplines. Experts included in the preliminary list were requested to suggest additional names. A total of 26 experts from 21 countries across the world were invited to participate through an email explaining the objectives and methodology of the project. Written consent from the experts was obtained to participate in two rounds of the survey and a virtual meeting. Every expert provided written consent to be acknowledged by name and affiliation in the manuscript.

### Delphi process

The modified Delphi process was implemented over two rounds of online surveys created using REDCap (Research Electronic Data Capture) web application hosted at IARC [12, 13]. Online surveys were piloted internally within IARC before being launched. The hyperlink was automatically generated and sent by REDCap to each expert to complete the online survey in an anonymized way.

Experts were furnished with the complete list of consolidated criteria from the systematic review for the first round of survey. They were requested to categorize each of them on a five-point Likert scale (strongly agree; agree; neutral; disagree; strongly disagree) as to whether the criterion would be considered essential (must have) for organized screening. The proportion of strongly agree and agree would be calculated among all experts. A definition of ‘consensus’ was set a priori in the protocol before launching the survey. ‘Consensus’ was considered to have been achieved when at least 80% of experts either strongly agreed or agreed on a particular criterion. In the first round of survey the experts were requested to suggest additional criteria that they felt could be added to the list. They were also given an option to rephrase any of the criteria to make them more legible or understandable without changing the core meaning.

Criteria identified as ‘essential’ at the first-round survey were dropped from the second-round survey, which requested the experts to select the ‘desirable’ (good to have) criteria from the remaining, using the same Likert scale. To maintain conformity between rounds, only those experts who responded to the first round were asked to respond to the second round. Again, consensus was considered to have been achieved when 80% or more of the experts agreed or strongly agreed upon a criterion being desirable. All the vote results and comments from the experts were recorded and stored in the REDCap.

Though no new essential criteria were suggested by the experts in the first round, some of them suggested rephrasing of selected criteria. Rephrasing of those criteria was done by PB and AC. Experts’ agreement (agree or do not agree) on the rephrased essential criteria was sought during the second round of survey, with the same predetermined 80% acceptance threshold. At each round, the experts had the option of expressing their opinion freely on the methodology of criteria selection and/or relevance of the criteria.

### Expert meeting

Experts participating in at least the first round of surveys were invited to a virtual meeting. The essential and desirable criteria identified based on the two rounds of the survey were presented at the meeting. Feedbacks were obtained from the experts, but they were not allowed to alter the criteria identified as essential or desirable through the Delphi process. Some experts requested us to define or clarify a few terms or phrases included in the criteria. These definitions or clarifications were discussed to arrive at a consensus. The proceedings of the meetings were recorded, and the recordings were used to note the experts’ feedbacks and suggestions, and the consent for recording the meeting was obtained at the start and before beginning recording.

## Results

A total of 70 articles, meeting the eligibility criteria of the systematic search, were included in the final analysis (Fig. 1). The systematic review yielded 69 criteria used by different authors to define cancer screening organization that were independently reviewed and listed by two experts (AC and PB). After removing the duplicates or the grossly overlapping ones, these criteria were consolidated into a final list of 32 (Additional file 2: Box S2).

Among the 26 experts who initially consented, 24 (92%) from 20 countries participated in both rounds of survey. The experts’ characteristics are listed in Table 1. Geographical representation and gender were reasonably balanced. Multi-disciplinarity in the field of cancer screening was achieved with expertise domains covering planning, management, evaluation and quality assurance of cancer screening programmes, cancer epidemiology and public health. More than half of the experts (*N*=14, 58%) had cancer screening related experience exceeding 20 years.

**Table 1:** Characteristics of the experts (*N*=24) participating in both rounds survey

Characteristics	***n*** (%)
**Gender**	
Female	10 (42)
Male	14 (58)
**Continents**	
Asia	4 (17)
Africa	2 (8)
Central and South America	5 (21)
Europe	7 (29)
North America	5 (21)
Oceania	1 (4)
**Current affiliation type (primary affiliation only)**	
National academic/research/public health institution	12 (50)
International academic/research/public health organization	7 (29)
Ministry of Health/Health Authority	3 (13)
Other	2 (8)
**Key areas of expertise (multiple responses allowed)**	
Cancer epidemiology/public health	22 (92)
Planning and management of cancer screening programme	17 (71)
Providing cancer screening related services	9 (38)
Cancer screening evaluation/quality assurance	19 (79)
Other	3 (13)
**Years of experience related to cancer screening**	
<10 years	3 (13)
10–19 years	7 (29)
20–29 years	8 (33)
30 years or more	6 (25)

In the first round, 16 out of 32 criteria were identified as ‘essential’ for organized cancer screening without any new ones being proposed. A total of seven essential criteria were rephrased, out of which four were accepted by the experts (Additional file 3: Table S1) and the rest were kept unchanged. Out of the 16 criteria not included among the essential ones, the experts selected eight as ‘desirable’ for organized screening.

### Essential criteria selected by the experts

The essential criteria are listed in Table 2. The two essential criteria showing the highest level of agreement of 96% (23/24) among experts were: having a protocol and/or guideline describing at least the target population, screening intervals, screening tests, referral pathway and management of positive cases; and a system of identifying the target population. The next three most agreed criteria (92% agreed, 22/24) were a system being in place for inviting eligible individuals for screening; having a policy framework from the health authorities defining governance structure, goals and objectives of the programme; and performance of screening programme being evaluated with appropriate indicators. The rest of the criteria had agreements ranging between 83% and 88%.

**Table 2:** List of essential criteria that define *organized cancer screening* identified by 24 experts, ranked by the proportion of experts strongly agree or agree

Essential criteria selected through Delphi round 1	% (strongly agree/agree)
1. Cancer screening programme has a protocol/guideline describing at least the target population, screening intervals, screening tests, referral pathway, management of positive cases^a^	96
2. There is a system in place for identifying the target population	96
3. There is a system in place for inviting eligible individuals for screening	92
4. Cancer screening programme has a policy framework from the health authorities defining governance structure, financing, goals and objectives of the programme^a^	92
5. Performance of screening programme should be evaluated with appropriate indicators	92
6. The protocol/guideline should at least describe: monitoring and evaluation	88
7. There is a system in place for notifying the results and informing about follow up	88
8. There is a system in place for sending recall notice to the non-compliant individuals	88
9. Auditing of the programme	88
10. A specified team/organization is responsible for quality assurance/ improvement	88
11. Performance of cancer screening programme is evaluated, published and widely disseminated on a regular basis^a^	88
12. All activities along the screening pathway are planned, coordinated and evaluated through a quality improvement framework (quality assurance)	88
13. An evidence-based protocol/guideline developed in consensus with majority of stakeholders	83
14. An information system exists with appropriate linkages (between population databases, screening information, cancer registry, etc.) for screening implementation and evaluation	83
15. The screening programme has a provision of continued training for service providers^a^	83
16. Performance of screening programme should be evaluated with reference standards for the indicators	83

^a^The criteria have been rephrased from their original versions and the rephrased version was accepted by the experts

### Desirable criteria selected by the experts

The desirable criteria are listed in Table 3. All except one (23/24, 96%) of the experts voted in favour of a specified organization or a team being responsible for programme implementation and/or coordination as a desirable criterion. The second most agreed upon (92% agreed, 22/24) desirable criterion was the health care professionals complying with protocol/guideline of the screening programme while delivering services.

**Table 3:** List of desirable criteria that define *organized cancer screening* identified by 24 experts, ranked by the proportion of experts strongly agree or agree

Desirable criteria selected through Delphi round 2	% (strongly agree/agree)
1. A specified organization or a team is responsible for programme implementation and/or coordination	96
2. Health care professionals comply with protocol/guideline of the screening programme while delivering services^a^	92
3. Cancer screening programme has a system in place to identify cancer occurrence in the target population (e.g. population-based cancer registry)^a^	88
4. The eligible individuals should be given informed choice with information on benefits and harms	88
5. The screening programme has an operational plan to encourage participation of the target population through improved awareness^a^	88
6. Appropriate legal framework exists for registration of individuals and establishing data linkages	83
7. Availability of adequate infrastructure, workforce and supplies for delivery of screening, diagnosis and treatment services	83
8. Equity of access to screening, diagnosis and treatment services should be built into the programme	83

^a^The criteria have been rephrased from their original versions

### Outcomes of the expert meeting

Out of 24 experts participating in the survey 21 attended the virtual plenary meeting, during which none of the experts raised any issue with the selection and categorization of the criteria. However, many of them raised concerns about the applicability of such criteria in the real programmatic settings, especially in the low- and middle-income countries (LMICs). Some of them expressed the need for further guidance on how to apply such criteria in settings where opportunistic screening is highly prevalent or programmes that are primarily implemented by the private sector or civil society organizations. The opinion of the experts was divided on whether a screening programme could be scored based on the number of criteria fulfilled. Though some experts felt that this would be a more objective way of documenting how far the programmes were in the process of being organized, no consensus was achieved. There was a general agreement that the list of essential and desirable criteria would be a good guidance for the programme managers to prioritize the improvement of services and negotiate with high-level policy-makers for resource allocation.

The terms and phrases further defined or clarified were auditing, policy framework, training of service providers and legal framework. We presented before the experts the definitions abstracted from various publications as starting points for discussion [14, 15]. The final definitions accepted are shown in Table 4.

**Table 4:** Definition or explanation of selected terms and phrases finalized with help from the experts

**A policy framework** (included in essential criteria no. 4) from the implementing organization (which may be governmental or non-governmental) defines the financial support, governance structure, goals and objectives of the screening programme to guide implementation and evaluation. It should describe the cooperation and the relationships between the stake-holders involved in the preparation, decision-making and implementation of the screening programmes. **Auditing** (included in essential criteria no. 9) is performed by sampling some or for all screening histories from the entire target population to identify the following groups of ‘screening failures’: • Cancers occurring in individuals who were not screened within the recommended interval • Cancers occurring in individuals who were screened and found to have an abnormality, but were not appropriately managed • Individuals who were adequately screened within the recommended interval with apparently normal results but developed cancer prior to next screening round. Cancers occurring outside target age group, overtreatment or screening related complications also need to be considered with the framework of auditing. **Continued training** (both knowledge-based and skill-based) (included in essential criteria no. 15) is ensured by the screening programme for all personnel involved in the screening pathway, including periodic refresher training and the supervisory support for new health providers. Such training can be provided by the programme or other stakeholders and is also regularly monitored. The service providers need regular feedbacks on their performance. **The legal framework** (included in desirable criteria no. 6) provides a legal mandate to the appropriate data protection safeguards and recognizes that a balance between fundamental rights of privacy and access to health services is crucial. The regulation of personal data safety, cancer screening registration, and the linkage between screening related data and other relevant data sources is necessary for effective screening management	

## Discussion

Using a systematic approach supported by the opinion of leading global experts in cancer screening, our study has built an international consensus on the essential and desirable criteria for a cancer screening programme to be considered as organized. This consensus would guide programme managers to implement quality-assured screening and facilitate policy makers to prioritize resource allocation. Traditionally, an organized programme is considered within the framework of a nationalized health system in which a governmental organization (regional or national) plans, implements, monitors, and funds activities across the entire screening continuum [16, 17]. The intrinsic value proposition for such a programme is that it would ensure equity of access to screening as well as downstream management and maximize health benefits through rigorous quality assurance at all levels. However, considering the significant variations in administration and delivery of screening programmes across the globe in different settings we need to broaden our vision and review the real-world value of a dichotomous labelling of screening programmes (organized vs. non-organized). This is also an opportunity to bring consistency in the use of various terminologies to describe screening programmes.

Recent publications from IARC have defined population-based screening programme as one with a mechanism to identify the eligible individuals and send personal invitations to them to attend screening [8, 9, 18, 19]. As our study has indicated, being population-based is one of the essential criteria for a programme to be considered as organized, though many times these terms (organized and population-based) are used as synonymous to each other. Quite often opportunistic screening is defined in opposition to organized screening, which was defined as screening activities occurring outside population-based programme as a result of a recommendation made by a health-care provider during a routine medical consultation, or by self-referral of individuals [18, 19].

Even though a few studies or guidelines have listed some of the elements of organized programme in the context of cervical cancer screening [20–22], there is no universally accepted definition of organized screening programme, which is the root cause of considerable confusion among those responsible for planning, implementation, and quality improvement of screening programmes. William et al conducted a review including 154 peer-reviewed articles on cervical cancer screening published between 1970 and 2014, which arrived at the conclusion that understanding of the term organized screening was quite variable and the programmes claiming to be organized differed significantly from prescribed norms and from each other [10]. Another more obvious example is the latest (2020) global report on non-communicable diseases (NCD) capacity assessment survey published by WHO [23]. Based on self-reporting by the NCD focal points within each country’s Ministry of Health the survey estimated that 40% of countries in the world had organized, population-based cervical cancer screening programmes, which was clearly an over-estimate. It is difficult to accept that 15% for low-income, 20% for lower-middle income and 50% for the upper-middle income countries have invitation-based screening programmes that fulfil other elements of organized screening. We matched the programme status reported by the participants of the WHO NCD survey with more detailed information collected by us from the Ministry of Health through the CanScreen5 project. Many of the programmes claiming to be organized population-based neither had a system of invitation nor fulfilled many of the criteria of organized screening.

There is another important reason why more clarity is required in our understanding of organized screening. The earliest concepts of organized screening were dominated by the Nordic- European style of implementing cervical cancer screening in the public sector, which was subsequently endorsed by the European cancer screening guidelines [24, 25]. However, the global eco-system for delivery of cancer screening is rapidly changing with private sector, not-for-profit organizations or health insurance companies managing screening programmes with some key elements of organized screening (e.g. registration of data, call-recall system and quality assurance) incorporated. Examples of such cancer screening programmes are those implemented by Kaiser Permanente in the USA, Pink Ribbon Red Ribbon Partnerships in several African countries, or Project ROSE in Malaysia [26]. In some of the sub-Saharan African countries cervical cancer screening is offered to women living with HIV at the anti-retroviral therapy clinics. Such a programme targeting high-risk population only is a combination of invitation-based (through community health workers) and opportunistic screening and is well-monitored by a central organization [27]. Some of the LMICs like Bangladesh or Morocco have shown that high volume opportunistic cancer screening programmes may be implemented incorporating at least some of the elements of organized screening [28, 29]. It is important to recognize the breadth of screening organization in practice and provide appropriate guidance to the opportunistic programmes on how best to organize cancer screening to derive maximum impact.

**Fig. 2:**
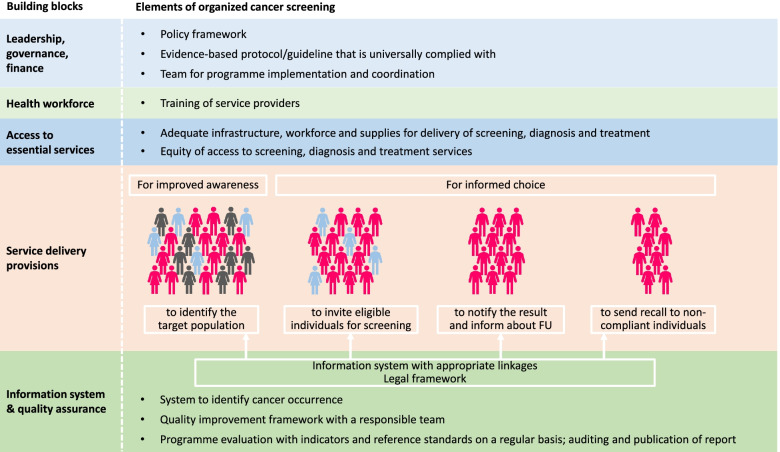
Building blocks for core elements of an organized screening programme

Due to such complexities in programme implementation and extreme heterogeneity in programme organization, it may be futile or even unfair to label screening programmes dichotomously as either organized or non-organized. The essential and desirable criteria listed in our study may be more productively used by the programme managers as a guide for continuous quality improvement and to set priorities for resource allocation. Programme managers can use the list of criteria and improve their programmes by incorporation of more essential or desirable elements in future planning. These are the norms that all screening programmes may aspire to achieve but certainly with variable levels of success, given the resource constraints or the parameters of existing healthcare systems. Improved organization of cancer screening requires reflection on and strengthening of health systems. The core elements of an organized screening programme described by our study as essential or desirable (Fig. 2) effectively create a framework for the building blocks of health system across cancer screening care cascade [30]. The essential and desirable criteria are fundamentally the various dimensions of health system building blocks as described below:

- *Leadership, governance, and financing for cancer screening*: A policy framework exists that describes the governance structure, programme objectives, financial resource allocation etc.; an evidence-based protocol and guideline exists; a team or organization is responsible for programme implementation, coordination, and protocol compliance.
- *Health workforce*: Service providers are adequate in number, and they have adequate provision for training and periodic reorientation.
- *Access to essential services*: Adequate infrastructure, workforce and supplies are available for seamless service delivery to all eligible individuals without causing financial hardships to them.
- *Service delivery*: Provision is there for population education to improve awareness; screening, diagnostic, treatment and follow up services are provided following the protocol and guideline; a system is in place for invitation of the eligible population and recalling those requiring further assessment/treatment. Every individual has informed choice.
- *Information systems and quality assurance*: A robust health information system with an appropriate legal framework exists so that it is capable of implementing invitation and call-recall and also capture performance data for programme evaluation with full respect for privacy legislation and ethical and deontological concerns; a team or organization is responsible for implementing quality improvement using appropriate indicators and standards.

Strengthening health systems across these building blocks will by itself lead to a better organized screening programme. Health system strengthening in the context of cancer screening requires a pragmatic selection of priorities based on available resources and competing health issues. This is often a gradual stepwise process synchronized with other health sector activities. The monitoring and evaluation framework of cancer screening needs to put equal stress on structural indicators (e.g. the number of health professionals providing screening for a defined population or the contribution of primary and secondary health care facilities delivering screening services) as much as is currently done on performance and outcome indicators. Such data usually collected through facility census will be more useful to monitor opportunistic screening programmes in the LMICs. In HICs (high-income countries) with a liberal health care system, health insurance databases and reimbursement policies based on scientific evidence may enable opportunistic screening programmes to adhere to several essential criteria of organized screening. Further research is needed to refine performance indicators. To allow benchmarking of programmatic efforts and to provide meaningful guidance on best practice in addressing quality and equity within cancer screening programme one may consider having a scoring system based on the essential and desirable criteria in the line of Index of Cancer Preparedness (ICP) [31]. The current convention of designating programmes as organized or non-organized is too simplistic and cannot be reliably applied to work in the current global landscape of cancer screening.

We acknowledge the limitation of our study. First, the intrinsic limitation of Delphi is its dependence on the composition of the experts (‘homogeneity in Consensus Group composition is likely to result in homogeneity of ratings’) [32]. We minimized such impact by ensuring our Delphi experts from different resource settings and in various fields of cancer screening. Besides, we set the threshold of agreement high, as at least 80% of experts strongly agree or agree. Next, due to the pandemic, the expert consensus meeting had to be held virtually, which limit the in-depth discussion to some extent compared to face-to-face meeting.

## Conclusions

To conclude, our study has listed all the elements that may be considered as best practices in organizing an impactful cancer screening programme. A well-functioning health system that is comprehensive, accessible, person-centred, well-coordinated with high accountability and efficiency is expected to achieve many of these elements, even if screening services are delivered in an opportunistic setting. Labelling programmes as organized or not is too simplistic, as does not provide much insight into the complex interplay of several determinants of success and best avoided.

## Supplementary Information

**Additional file 1: Box S1.** Search strategy of the systematic review for organized cancer screening programme.

**Additional file 2: Box S2.** The list of 32 consolidated criteria for organized cancer screening programme from the systematic review.

**Additional file 3: Table S1.** Experts’ voting result on modified essential criteria.

## Data Availability

The datasets generated and/or analysed during the current study are included within the article and the supplementary information.

## Abbreviations

CanScreen5: Cancer Screening in Five Continents
HICs: High-income countries
IARC: International Agency for Research on Cancer
ICP: Index of Cancer Preparedness
LMICs: Low- and middle-income countries
NCD: Non-communicable diseases
REDCap: Research Electronic Data Capture
WHO: World Health Organization
