# Supplementary material for: An international consensus on the essential and desirable criteria for an ‘organized’ cancer screening programme
Source: BMC Med. 2022 Mar 23;20:101. doi: 10.1186/s12916-022-02291-7 (PMC8941752; doi:10.1186/s12916-022-02291-7)
Supplement: Supplementary file 1 — Additional file 1: Box S1. Search strategy of the systematic review for organized cancer screening programme. [file 12916_2022_2291_MOESM1_ESM.pdf]

Box S1: Search strategy of the systematic review for organized cancer screening programme

- Databases: PubMed
- Search string
  1. neoplasms[MH] OR cancer*[tiab] OR neoplas*[tiab] OR tumor*[tiab] OR tumour*[tiab] OR malignan*[tiab]
  2. mass Screening[MH] OR early detection of cancer[MH]
  3. screening*[tw] OR early detect*[tw]
  4. #2 OR #3
  5. #1 AND #4
  6. organized[tiab] OR organised[tiab] OR population-based[tiab] OR population based [tiab]
  7. program[tiab] OR programs[tiab] OR programme[tiab] OR programmes[tiab]
  8. #6 AND #7
  9. #5 AND #8
  10. Limit 9 to (books and documents or comment or editorial or guideline or legislation or letter or practice guideline or review or systematic review or technical report)

- Databases: Web of Science Core Collection
- Search string
  1. TS=(neoplasm) OR TI=(cancer*) OR AB=(cancer*) OR TI=(neoplas*) OR AB=(neoplas*) OR
  2. TI=(tumor*) OR AB=(tumor*) OR TI=(tumour*) OR AB=(tumour*) OR TI=(malignan*) OR AB=(malignan*)
  3. TS=(screening*) OR TS=(early detect*)
  4. #1 AND #2
  5. TI=(organized) OR AB=(organized) OR TI=(organised) OR AB=(organised) OR TI=(population-based) OR AB=(population-based) OR TI=(population based) OR AB=(population based)
  6. TI=(program) OR AB=(program) OR TI=(programs) OR AB=(programs) OR TI=(programme) OR AB=(programme) OR TI=(programmes) OR AB=(programmes)
  7. #4 AND #5
  8. #3 AND #6
  9. Limit 8 to (review or editorial material or letter or book chapter)

- Databases: Embase
- Search string
  1. neoplasm/
  2. “cancer*” ab.ti.
  3. “neoplas*” ab.ti.
  4. “tumor*” ab.ti.
  5. “tumour*” ab.ti.
  6. “malignan*” ab.ti.

Box S1: Search strategy of the systematic review for organized cancer screening programme

7. #1 or #2 or #3 or #4 or #5 or #6
8. mass screening/
9. early cancer diagnosis/
10. “screening*” tw.
11. “early detect*” tw.
12. #8 or #9 or #10 or #11
13. #7 and #12
14. (organized or organized or population-based or population based).ab.ti.
15. (Program or programs or programme or programmes).ab.ti.
16. #14 and #15
17. #13 and #16
18. Limit 17 to (books or chapter or editorial or letter or “review”)

- Databases: PubMed Bookshelf
- Search string

1. cancer[title]
2. screening [title]
3. #1 and #2

•Inclusion and exclusion criteria:

To be included in the systematic review, eligible studies were: 1) about cancer screening; 2) intended to discuss aspects of cancer screening programme implementation; 3) with three or more characteristics/definition/criteria of organized programmes. We excluded studies if: 1) they were not related to cancer screening; 2) studies only on efficacy, effectiveness, safety, acceptability of cancer screening tools and technology; 3) less than three characteristics/definition/criteria of organized programmes.
