# Supplementary material for: An international consensus on the essential and desirable criteria for an ‘organized’ cancer screening programme
Source: BMC Med. 2022 Mar 23;20:101. doi: 10.1186/s12916-022-02291-7 (PMC8941752; doi:10.1186/s12916-022-02291-7)
Supplement: Supplementary file 2 — Additional file 2: Box S2. The list of 32 consolidated criteria for organized cancer screening programme from the systematic review. [file 12916_2022_2291_MOESM2_ESM.pdf]

Box S2: The list of 32 consolidated criteria for organized cancer screening programme from the systematic review

1. A policy framework defining the goals and objectives of the programme
2. An evidence-based protocol/guideline developed in consensus with majority of stakeholders
3. The protocol/guideline should at least describe: the target age, screening intervals, screening tests and management of positive cases
4. The protocol/guideline should at least describe: *monitoring and evaluation*
5. Availability of adequate infrastructure, workforce and supplies for delivery of screening, diagnosis and treatment services
6. Dedicated funding/budget to ensure delivery of following services free of cost (may be reimbursable) to the eligible individuals screening services
7. Dedicated funding/budget to ensure delivery of following services free of cost (may be reimbursable) to the eligible individuals diagnostic services
8. Dedicated funding/budget to ensure delivery of following services free of cost (may be reimbursable) to the eligible individuals treatment services
9. Screening is administered as a public service by a government body
10. A specified organization or a team is responsible for programme implementation and/or coordination
11. Professionals involved comply with the national protocol/guideline
12. Appropriate legal framework exists for registration of individuals and establishing data linkages
13. An information system exists with appropriate linkages (between population databases, screening information, cancer registry, etc.) for screening implementation & evaluation
14. There is a system in place for identifying the target population
15. There is a system in place for inviting eligible individuals for screening
16. There is a system in place for notifying the results & informing about follow up
17. There is a system in place for sending recall notice to the non-compliant individuals
18. There is provision for training of service providers
19. Programme should have high participation/coverage
20. All activities along the screening pathway are planned, coordinated and evaluated through a quality improvement framework (quality assurance)
21. Auditing of the programme
22. A specified team/organization is responsible for quality assurance/improvement
23. Performance of screening programme should be evaluated with appropriate indicators
24. Performance of screening programme should be evaluated with reference standards for the indicators
25. Performance of screening programme should be evaluated with appropriate indicators and published regularly
26. A system should be in place for identifying cancer occurrence in the target population
27. There should be documented strategies for increasing awareness of the population to encourage participation

Box S2: The list of 32 consolidated criteria for organized cancer screening programme from the systematic review

28. The eligible individuals should be given informed choice with information on benefits and harms
29. There should be an informed consent
30. Equity of access to screening, diagnosis and treatment services should be built into the programme
31. Effectiveness and cost-effectiveness of screening should be demonstrated through health technology assessment in the country
32. Piloting of the screening programme should be done before scaling-up
