# Supplementary material for: An international consensus on the essential and desirable criteria for an ‘organized’ cancer screening programme
Source: BMC Med. 2022 Mar 23;20:101. doi: 10.1186/s12916-022-02291-7 (PMC8941752; doi:10.1186/s12916-022-02291-7)
Supplement: Supplementary file 3 — Additional file 3: Table S1. Experts’ voting result on modified essential criteria. [file 12916_2022_2291_MOESM3_ESM.docx]

**Table S1: Experts’ voting result on modified essential criteria**

| **Original Components** | **Modified components** | **% agree** |
| --- | --- | --- |
| The protocol/guideline should at least describe: the target age, screening intervals, screening tests and management of positive cases | Cancer screening programme has a protocol/guideline describing at least the target population, screening intervals, screening tests, referral pathway, management of positive cases | 96 |
| There is provision for training of service providers | The screening programme has a provision of continued training for service providers | 96 |
| A policy framework defining the goals and objectives of the programme | Cancer screening programme has a policy framework from the health authorities defining governance structure, goals and objectives of the programme | 88 |
| Performance of screening programme should be evaluated and published regularly | Performance of cancer screening programme is evaluated, published and widely disseminated on a regular basis | 88 |
| Auditing of the programme | Cancer screening programme is audited and the report is made public | 79 |
| Performance of screening programme should be evaluated with appropriate indicators | Performance of cancer screening programme is evaluated with indicators that are internationally recognized and adopted | 71 |
| Performance of screening programme should be evaluated with reference standards for the indicators | Indicators used to evaluate performance of cancer screening programme are locally defined and ideally, evidence-based reference standards | 67 |
